# The Influence of the Drawing Process on the Mechanical Properties of TRIP Steel Wires with 0.4% C Content

**DOI:** 10.3390/ma13245769

**Published:** 2020-12-17

**Authors:** Monika Kucharska, Sylwia Wiewiórowska, Jacek Michalczyk, Andrzej Gontarz

**Affiliations:** 1 Independent Researcher, ul. Bałtycka 9/11, 42-202 Czestochowa, Poland; mkucharska.priv@gmail.com; 2 Faculty of Production Engineering and Materials Technology, Czestochowa University of Technology, 19 Armii Krajowej Av., 42-200 Czestochowa, Poland; sylwia.wiewiorowska@pcz.pl; 3 Faculty of Mechanical Enginiering, Lublin University of Technology, 36 Nadbystrzycka Av., 26-218 Lublin, Poland; a.gontarz@pollub.pl

**Keywords:** multiphase steel, TRIP steel, wires, drawing process

## Abstract

In the work, the results of the research concerned with the TRIP (Transformation Induced Plasticity) steel wire drawing process in experimental and theoretical ways are shown. The wire drawing process tests on the experimental way were conducted in both laboratories as well as industrial conditions, with the use of two drawing speeds (1.6 and 6 m/s) and two drawing schemes (low and high single reductions). The mechanical properties of wires drawn with high drawing speed equal to 6 m/s showed higher values of mechanical properties for wires drawn with low single reductions than for wires drawn with high single reductions. Such a phenomenon contradicts the theory of drawing wires from steel with a ferritic-pearlitic structure and must be related to TRIP structure and the presence of retained austenite in it, which is transformed into martensite during the deformation process. In order to explain this phenomenon, the theoretical wire drawing process analysis was conducted with the use of the Drawing 2D program based on the finite element method. On the base of the simulation, a large increase in temperature was found on the surface for wires drawn with high drawing speed and low single reductions, which can cause the blocking of transformation retained austenite into martensite and thus a decrease in R_m_. To confirm this thesis, further studies will include tests of the amount of retained austenite in wires obtained during experimental tests.

## 1. Introduction

Significant progress in metallurgical technologies allows the production of different grades of steels from typical ferritic steels to multiphase high-strength steels, which are new materials having high mechanical properties while retaining very good plastic properties. An example of this type of materials is TRIP steels, which are currently used in the automotive industry for car body sheet in the controlled crash zone construction. TRIP steels are classified into the group of advanced high strength steels, which are distinguished by very high mechanical properties (above 700 MPa, reaching even 2000 MPa) and the percentage elongation being contained in a fairly wide range from 5 to 30%. Many publications concerned with the TRIP effect, the technology of producing TRIP steel sheet and its applications were found to exist in available literature; however, there were no publications concerning the technology of producing wire rod of low- and medium-carbon TRIP-structure steels and research on the drawing process and properties of wires of this type of steel. Therefore, in 2005, a team of researchers from the Drawing Engineering and Metal Products Department at the Czestochowa University of Technology, which included the authors of this paper, undertook studies on this subject matter, as the first on the global scale [1,2,3,4,5,6,7,8].

A factor determining the advantageous properties of the TRIP steel is the retained austenite contained in its structure, which undergoes a transformation into martensite during plastic deformation; this phenomenon is termed the transformation induced plasticity (TRIP) effect [9,10,11]. The martensitic transformation may occur under the influence of stresses, strains or temperature [12].

Multiphase TRIP structure containing ferrite, bainite, and retained austenite we can obtain by carrying out two-stage heat treatment process which consisted of holding the steel within the dual-phase austenitic–ferritic range with subsequent soaking it within the bainitic transformation range.

Studies reported in the literature show that for low-carbon steel (to 0.1% C), it possible to obtain from 8 to 10% of retained austenite [13]. Limiting carbon content for TRIP steel oscillates around the 0.4% value because the increased volume fraction of carbon content can lead to excessive stabilization of retained austenite, which can contribute to the blocking transformation of retained austenite, which decides about the TRIP effect.

According to De Cooman [14], the carbon content for TRIP steel can oscillate about values from 0.12 to 0.55%. Jacques and others stated that the steel with TRIP effect should have carbon content from 0.1 to 0.4% [15].

The TRIP steels with high retained austenite content, which undergo martensite transformation gradually, with strain increasing, are characterized by the best mechanical properties. This is the effect of presence in steel structure the mechanical stable retained austenite, which extends the plasticity range of material, and martensite obtained after transformation, influences on the increase of the mechanical properties.

In this work, the analysis of the influence of the parameters of the drawing process (drawing speed and the value of using single reductions) on the mechanical properties of drawn TRIP steel wires was carried out during experimental work. The research was conducted for TRIP steel with 0.43% of carbon content and with a high amount of retained austenite in the structure obtained after a two-step heat-treatment process.

In order to explain the atypical phenomenon of the increase in the mechanical properties of drawn wires with low single reductions in relation to wires drawn with high single reductions, a theoretical analysis of the drawing process was carried out.

## 2. Materials and Methodology

The study material comprised of a medium-carbon steel wire rod. The chemical composition of the tested steel was as follows (wt %): C—0.431%, Mn—1.470%, Si—1.363%, Cr—0.100%, V—0.001%, Al—0.050%, U—0.007%, Mo—0.001%, Ni—0.129%.

The conventional ferritic-pearlitic structure was obtained in the wire rod after the conventional hot rolling process. Therefore, in order to obtain the appropriate amount of retained austenite in the material, ensuring the TRIP effect in the steel, it was necessary to carry out a two-stage heat treatment with appropriately selected parameters established in previous research [16].

The two-stage heat treatment process making possible the obtaining TRIP structure was realized in laboratory conditions of the Technical University of Częstochowa, on LAC resistance heating furnaces (LAC, s.r.o., Židlochovice, Czech Republic).

**Figure 1:**
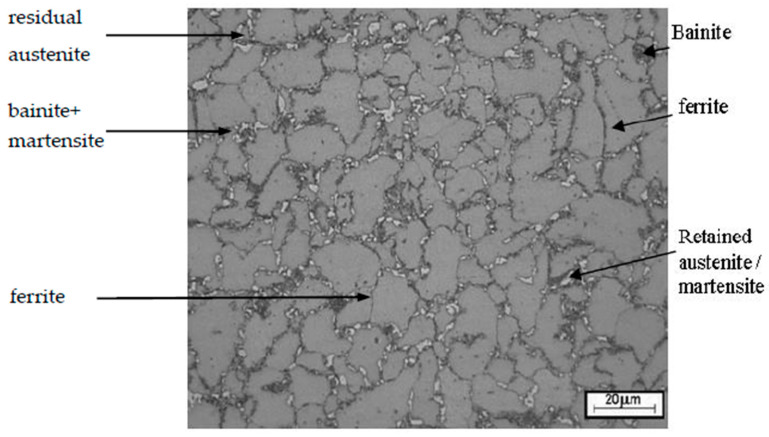
Transformation induced plasticity (TRIP) steel microstructure after a two-stage heat treatment conducted according to optimized process parameters as etched with the Lichtenegger and Blöch reagent (29% of retained austenite).

The microstructure of the wire rod intended for the studies was characterized by a multiphase TRIP-type structure, which consists of ferrite, bainite, few martensitic precipitations and retained austenite, the most important structural component, which determines the impact on steel properties. In the case of the materials used for the tests, the volume fraction of retained austenite within the wire rod structure after a completed two-stage treatment was approx. 29%, with the microstructural image shown in Figure 1.

## 3. Tests Involving TRIP Steel Drawing Process

A drawing process according to variants taking into account various process parameters was executed in order to conduct a complete analysis and determine the impact of a single drawing reduction degree and the drawing speed on the mechanical properties of TRIP steel wires with an 0.43% carbon content.

The wire rod was prepared for the cold drawing process by cleaning the surface of scale generated within the heat treatment process and applying a lime lubricant carrier.

The drawing process involving a TRIP steel wire rod with a diameter of 6.30 mm and a final diameter of 2.60 mm was conducted in laboratory and industrial conditions. A JP600 block drawing machine (ITALMEC Sp. z o.o., Katowice, Poland) was used in laboratory studies. The industrial tests were conducted for higher drawing speeds using a SAMP BS700 drawing machine (ITALMEC Sp. z o.o., Katowice, Poland). The process uses carbide conical drawing dies with a drawing angle of 2α = 12°. The drawing process was conducted for two schemes of drawing process, low and high single reductions, at two drawing speeds: V_1_ = 1.6 m/s and V_2_ = 6.0 m/s (Table 1, Table 2 and Table 3).

## 4. Tests Involving the Mechanical Properties of TRIP Steel Wires Drawn with Various Process Parameters

**Figure 2:**
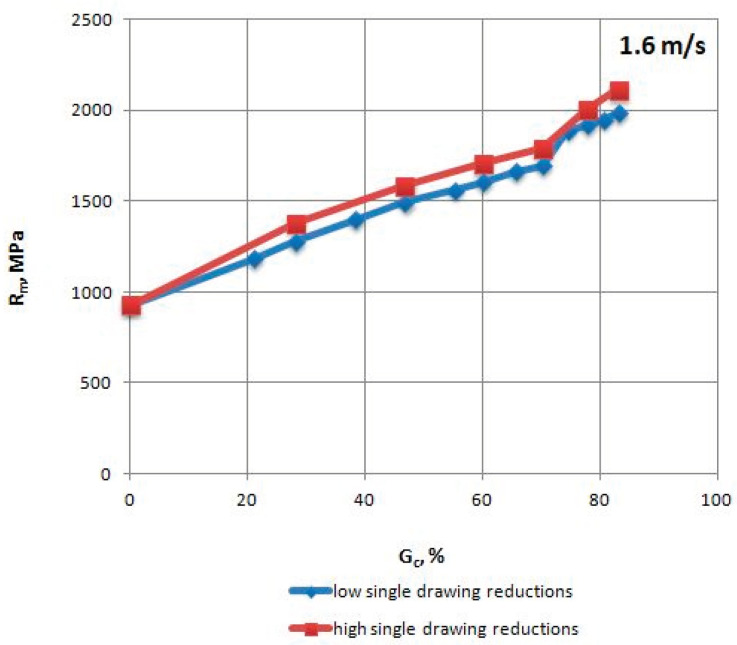
Change in the tensile strength R_m_ of TRIP steel wires for variants with low and high single drawing reductions, at a speed of 1.6 m/s, as the total drawing reduction function.

**Figure 3:**
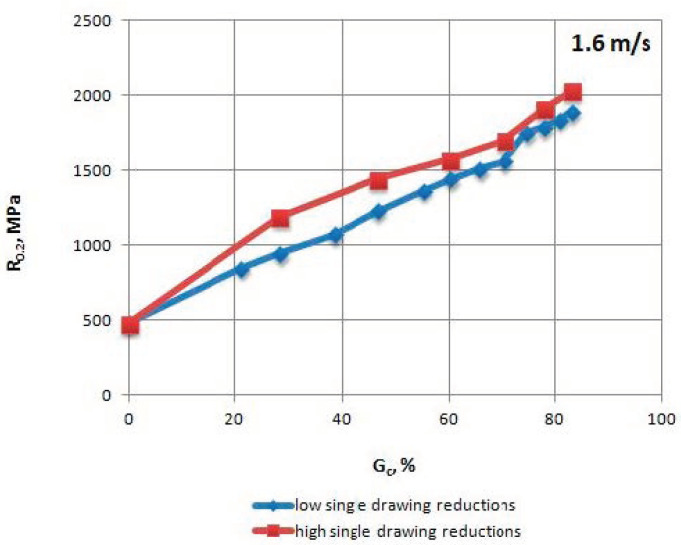
Change of the proof yield strength R_0.2_ of TRIP steel wires for variants with low and high single drawing reductions, at a speed of 1.6 m/s, as the total drawing reduction function.

**Figure 4:**
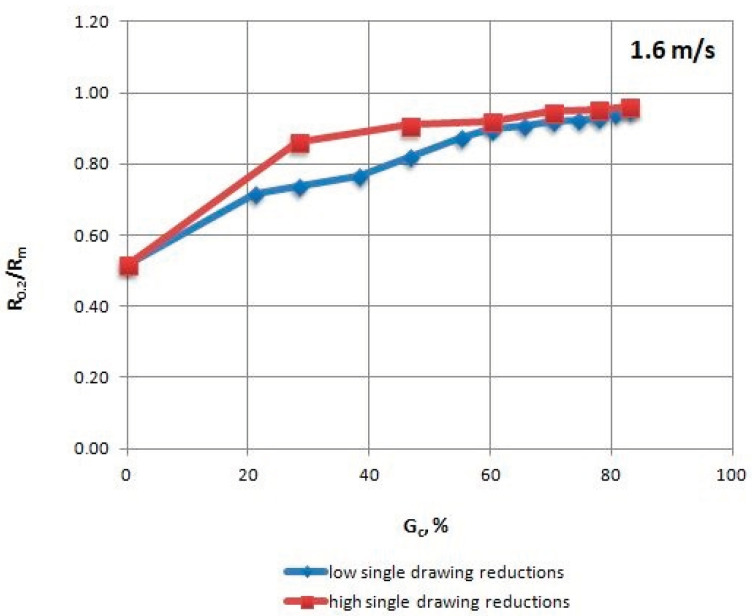
Change of the R_0.2_/R_m_ coefficient of TRIP steel wires for variants with low and high single drawing reductions, at a speed of 1.6 m/s, as the total drawing reduction function.

**Figure 5:**
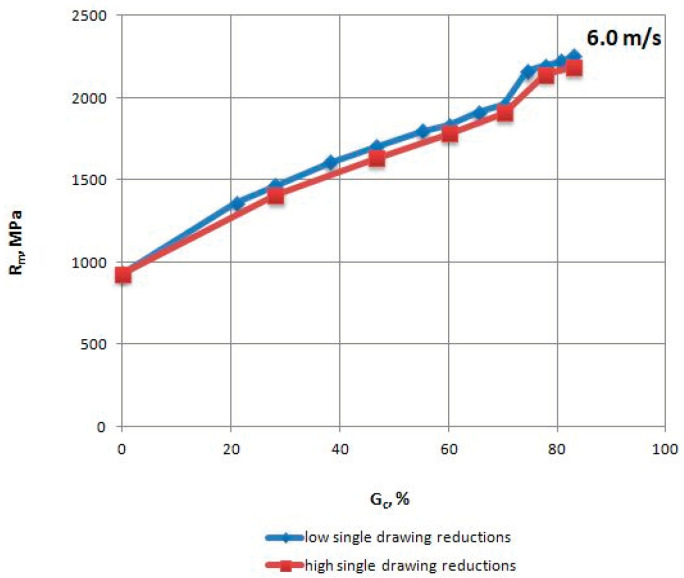
Change in the tensile strength Rm of TRIP steel wires for variants with low and high single drawing reductions, at a speed of 6.0 m/s, as the total drawing reduction function.

**Figure 6:**
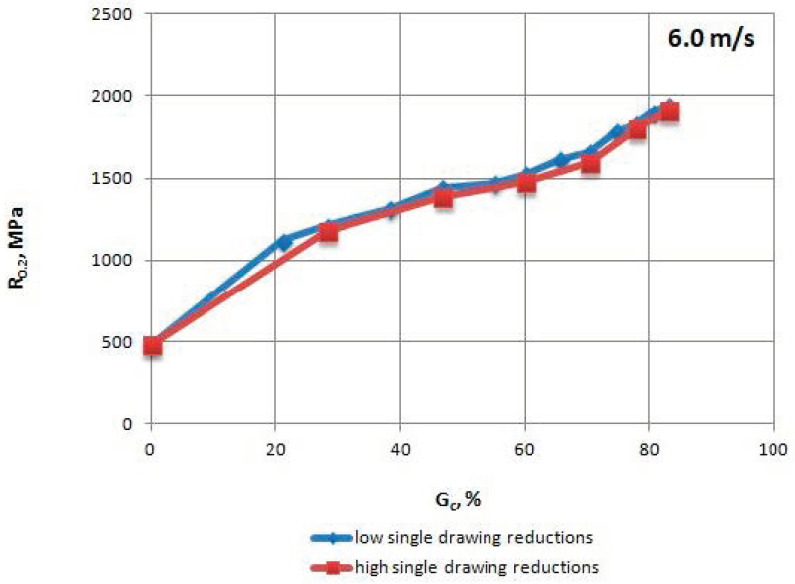
Change of the proof yield strength R_0.2_ of TRIP steel wires for variants with low and high single drawing reductions, at a speed of 6.0 m/s, as the total drawing reduction function.

**Figure 7:**
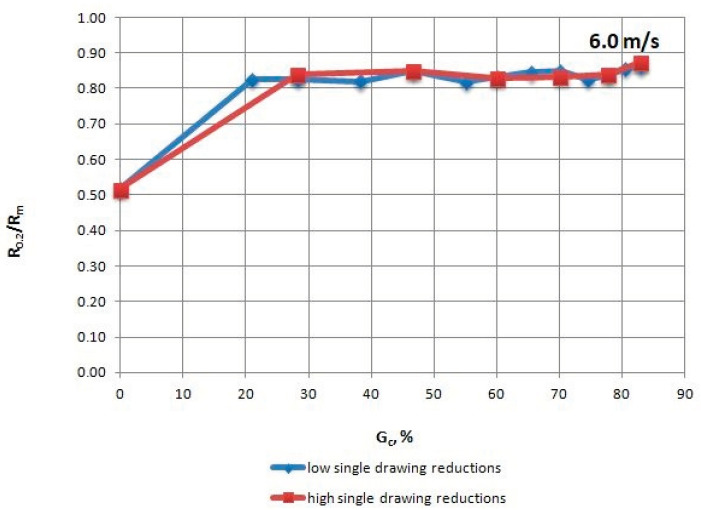
Change of the R_0.2_/R_m_ coefficient of TRIP steel wires for variants with low and high single drawing reductions, at a speed of 6.0 m/s, as the total drawing reduction function.

The mechanical properties of TRIP steel wires were tested according to different variants drawing process described in Table 1 after each draw. The research was conducted according to Polish standard PN-EN ISO 6892-1:2010 [17], on a ZWICK/Z100 testing machine (Zwick Roell Polska Sp. z o.o. Sp. K., Wrocław, Poland). The results are shown in Table 4, Table 5, Table 6 and Table 7 and Figure 2, Figure 3, Figure 4, Figure 5, Figure 6 and Figure 7.

Based on the test results shown in Figure 2 and Figure 3, it can be concluded that for a drawing speed of 1.6 m/s, the mechanical properties of wires (R_m_ and R_0.2_) drawn with high (approx. 25.5% on average) single drawing reductions are higher (after all process stages) than the properties of wire draw with low (approx. 13.7% on average) single reductions.

This phenomenon is typical when drawing wires with different single drawing reductions since when the value of single cross-section decrements increases in individual draws, the Rm of the wires also increases. This results from the greater strengthening of the material caused by increased non-dilatational strain in the subsurface layer of the wires.

When analyzing the change in the R_0.2_/R_m_ coefficient (Figure 4), higher plasticity of the wires drawn with low single reductions can be observed relative to wires with high single reductions, especially in the range of 20–50% of the total drawing reduction, when the difference reaches 19%.

Presumably, this is due to the fact that the speed of transformation of retained austenite into martensite is proportional to the strain level within a given draw, which is why a wire drawn with a single low reduction maintains larger quantities of retained austenite in its structure, which ensures its greater plasticity.

When analyzing the mechanical properties of wires drawn with different single cross-section decrements, at a drawing speed of every stage of Vc = 6.0 m/s, it was concluded that both R_m_ (Figure 5), as well as R_0.2_ (Figure 6), is higher for wires drawn with low single drawing reductions, relative to the ones drawn with high single reductions. It was also found that in the case of the 0–25% range of the total drawing reduction, there was a more rapid reduction in the plasticity (R_0.2_/R_m_) of wires drawn with low single drawing reductions (Figure 7).

This phenomenon contradicts the theory and technology of drawing steel wires with a typical ferritic-pearlitic structure; therefore, it must be associated with the TRIP structure of drawn wires and the transformation of austenite into martensite ongoing therein during heat treatment, which depends on the strain level and speed.

It was decided to conduct a theoretical process analysis in order to explain this phenomenon.

## 5. Drawing Process Theoretical Analysis

The theoretical analysis of the process of drawing TRIP steel wires was conducted using Drawing 2D software (version 2), which processes data based on the finite element method (FEM) [18].

Boundary conditions corresponding to the conditions encountered during experimental tests were assumed for the numerical simulation. Modeling was executed for the speeds of 1.6 and 6.0 m/s using low and high single drawing reductions shown in Table 1.

The drawing process was analyzed using classing conical dies with an angle of 2α = 12°. In the experimental analysis, TRAXIT CCF grease (Traxit Internatiional GmbH, Schwelm, Germany) was used as a lubricant in the drawing process, which reflects the semi-dry friction conditions.

Therefore, in numerical analysis, the value of friction coefficient (µ) between tool and material was estimated to 0.07.

The initial material temperature was 20 °C. The body deformation model was developed for the axial-symmetrical process and the conditions of the nonisothermic flow of an inaccuracy, rigid-plastic medium with nonlinear strengthening.

It is assumed that the friction conditions of Amontons–Coulomb are met on the surface of the die and wire. The contact and friction conditions between the material and the tool were modeled using the penalty function method.

Modeling such a process required a boundary task solution from the field of plastic processing theory taking into account heat flow, material heating from plastic deformation and friction, and transmission of data on material properties from string-to-string.

**Figure 8:**
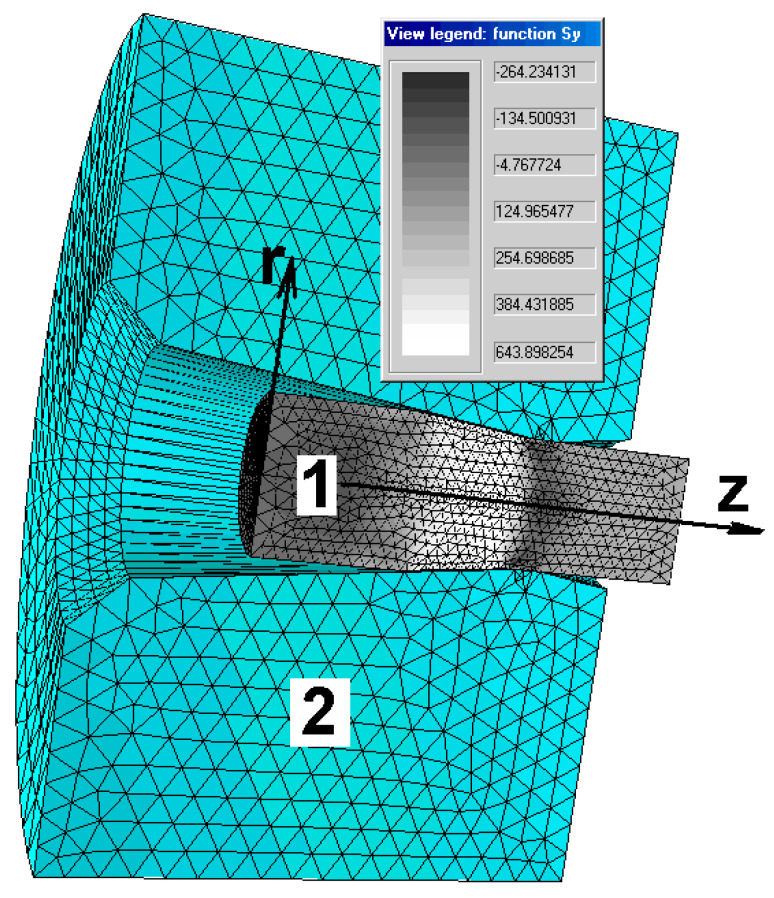
An example of a generated finite element mesh for drawing material (1) and die (2) and an example of a stress field distribution along the *z*-axis, marked in the computer program as *σ_y._*

In the program, during calculations according to this model, a system was introduced that automatically generates a mesh of finite elements for each string and takes into account the dimensions of the tool and material (entered by the user of the program) [18]. Isoparametric triangular finite elements were applied to the mesh (Figure 8).

The heat model was considered a quasi-static problem.

When solving the heat task, the passage of the separate section of material through the thrust drawing cone, step-by-step, with the solution of subsequent part-time tasks at each step of the transition, was considered.

**Figure 9:**
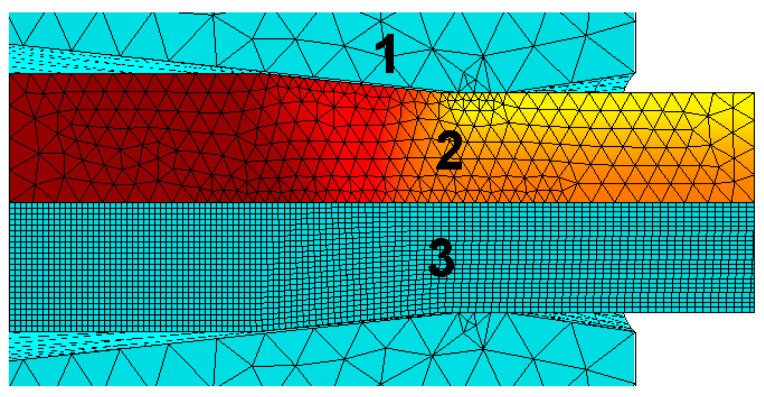
Mesh diagram for the heat-plastic solution and process visualization, 1—tool mesh, 2—deformation and plastic task mesh, 3—Lagrange mesh.

Lagrange’s rectangular mesh is also generated to visualize character strains in order to make the process easier to analyze. The displacement of each node of this mesh is calculated from the deformation of the “working” triangular mesh (Figure 9).

To determine the rheological model of the material that was introduced into the program database, plasticometric studies were conducted on the Gleeble 3800 thermomechanical simulator (Dynamic Systems Inc., El Segundo, CA, USA). The studies were conducted for four deformation speeds: 5.0 s^−1^, 70 s^−1^, 150 s^−1^ and 240 s^−1^, at four temperatures of 20 °C, 200 °C, 400 °C and 800 °C.

In order to obtain a mathematical relationship that makes the yield stress value ($\sigma_{p}$) dependent on deformation parameters, $\left(T,\varepsilon ,\overset{\cdot }{\varepsilon }\right)$ the results of the tests were approximated with the functional relationship specified by Henzel-Spittel [19], presented by Equation (1). The function parameters can be found in Table 8.

(1) $$\sigma_{p}=a_{1}\varepsilon^{a_{2}}e^{\left(\frac{a_{3}}{\varepsilon }\right)}e^{\left(a_{4}\varepsilon \right)}(1+\varepsilon {)}^{a_{5}T}{\dot{\varepsilon }}^{a_{6}}{\dot{\varepsilon }}^{a_{7}T}T^{a_{8}}e^{\left(a_{9}T\right)}$$

**Figure 10:**
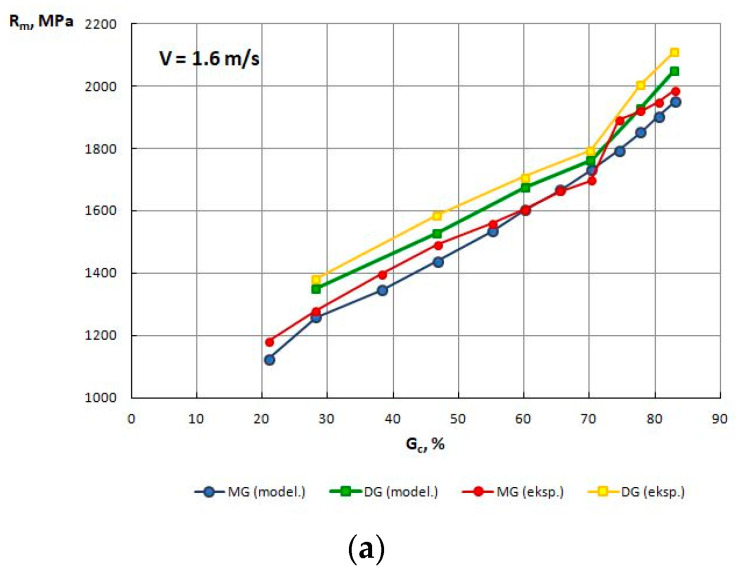

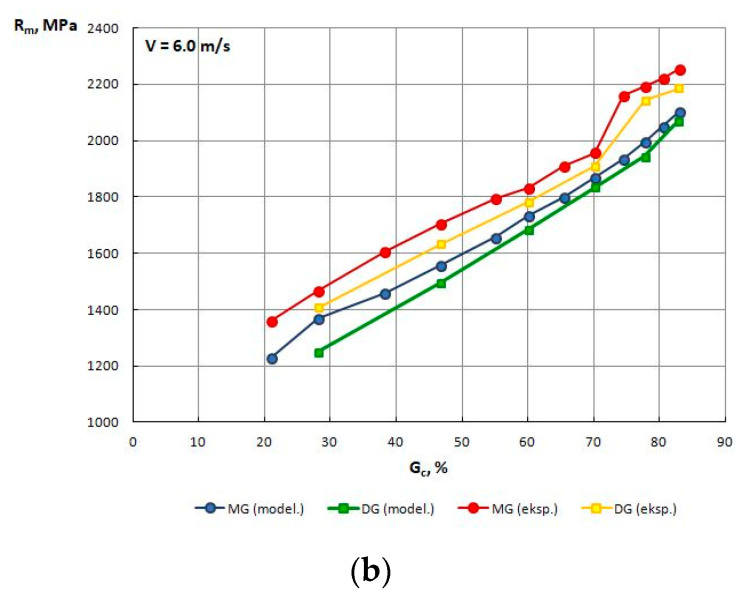
Change in the R_m_ of TRIP steel wires drawn with various speed, and low—MG and high—DG single drawing reductions as a function of the total drawing reduction G_c_%, obtained through numerical modeling and an experiment. (**a**) Speed drawing V = 1.6 m/s, (**b**) Speed drawing V = 6.0 mm/s.

The function parameters (1) in Table 8 were used in the computer simulation of the drawing process. Drawing 2D software, in addition to the ability to read the values of individual parameters from the nodes of the finite element mesh, also calculates the average value of Rm (for the entire wire cross-section) over individual strings. Therefore, Table 9 and Figure 10 show the tensile strength of the wires (drawn with different process parameters) obtained from the simulation and compared them with the Rm values obtained from the experimental tests presented in the previous chapter. The standard deviation of tensile strength values obtained from the experiment and theoretical analysis is around 10 MPa.

Based on the tests shown in Table 9 and Figure 10, it can be concluded that modeling results exhibit a good correlation with experimental results (generally, differences below 10%), which proves the correct rheology of the material entered in the Drawing 2D software.

The numerical modeling results also confirmed that for the drawing speed V_c_ = 6.0 m/s, the R_m_ in wires drawn with high single reductions was lower than the R_m_ in wires drawn with low single reductions.

The surface temperature of the wire rises sharply along the length of the die working cone of the die. This is due to the heat release of plastic deformation and friction. In the gauging cylinder of the die, we still observe a slight increase in the temperature of the wire caused by friction, while after exiting the die, the temperature of the wire drops.

The material temperature in the wire axis increases quite quickly along the length of the working cone of the die and stabilizes in the gauging cylinder of the die. This is due to the fact that the material temperature in the wire axis is not directly influenced by the heat generated by friction on the contact surface of the die and wire. After the wire exits the die, the temperature of the material along the axis begins to increase as the temperature equalizes between the surface and axis of the wire, which is caused by thermal conductivity.

Significant temperature differences between the surface of the wire and its axis result not only from the heat associated with friction on the surface but mainly from the nonuniform deformation, i.e., unnecessary non-dilatational strain occurring outside the axis of the material and increasing from the axis to the surface of the wire.

**Figure 11:**
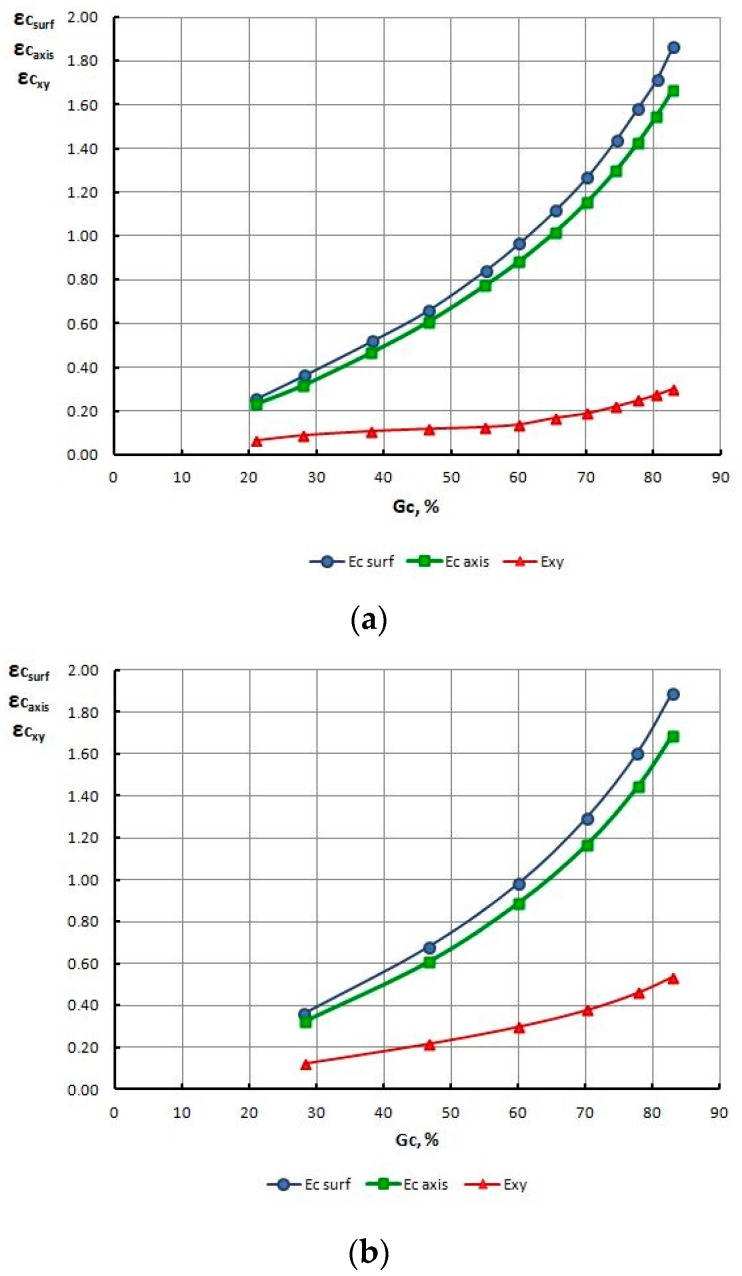
Change of strain level on the surface ε_c surf_, axis ε_c axis_ and non-dilatational strain ε_xy_ in wires drawn with low (**a**) and high (**b**) single section reductions, at a drawing speed of V_c_ = 1.6 m/s, as a total drawing reduction function.

**Figure 12:**
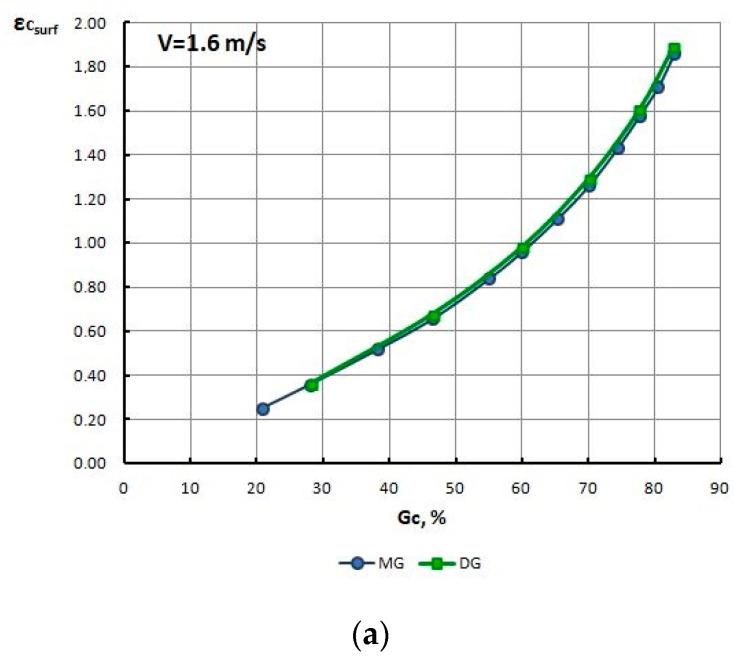

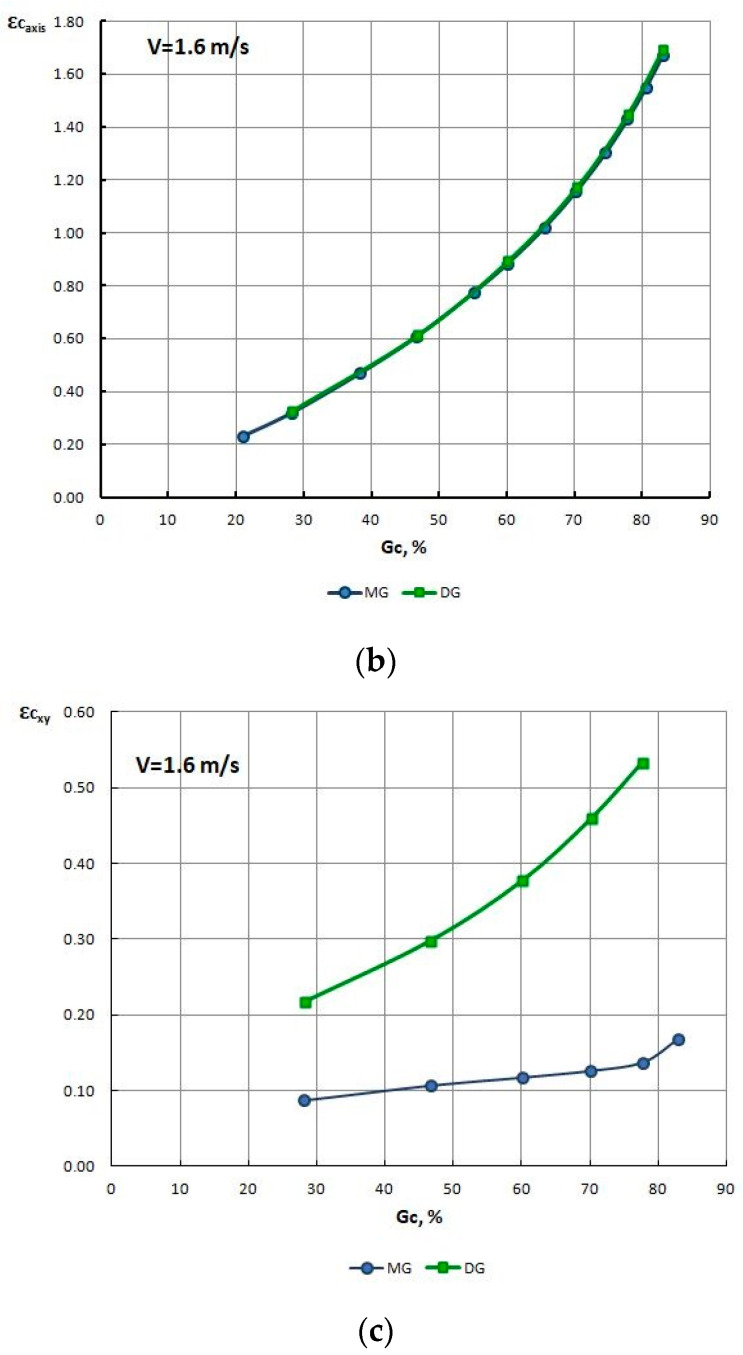
Change of strain level on the surface (**a**), axis (**b**) and non-dilatational strain (**c**) in wires drawn with low MG and high DG single section reductions, at a drawing speed of V_c_ = 1.6 m/s, as a function of the total drawing reduction G_c_%.

**Figure 13:**
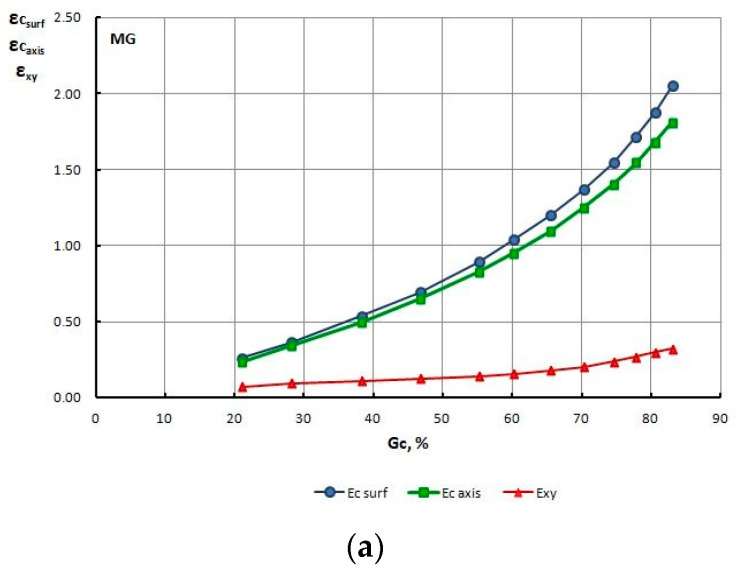

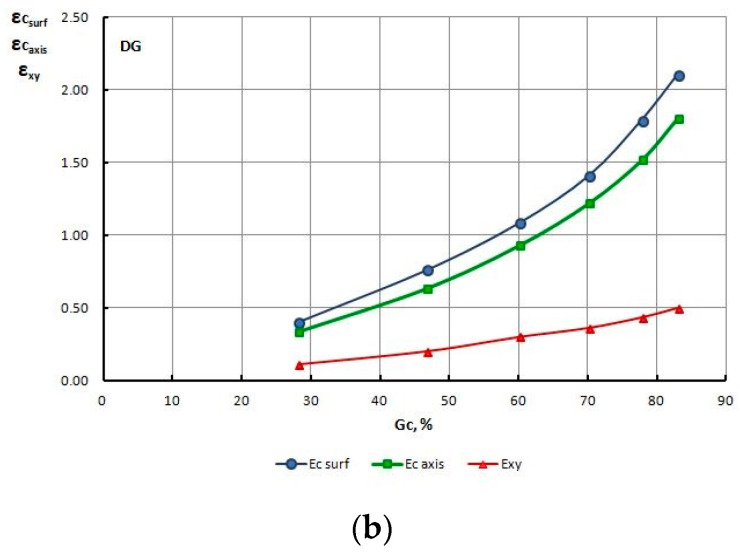
Change of strain level on the surface ε_c surf_, axis ε_c axis_ and non-dilatational strain in wires drawn with low (**a**) and high (**b**) single section reductions, at a drawing speed of V_c_ = 6.0 m/s, as a total drawing reduction function.

**Figure 14:**
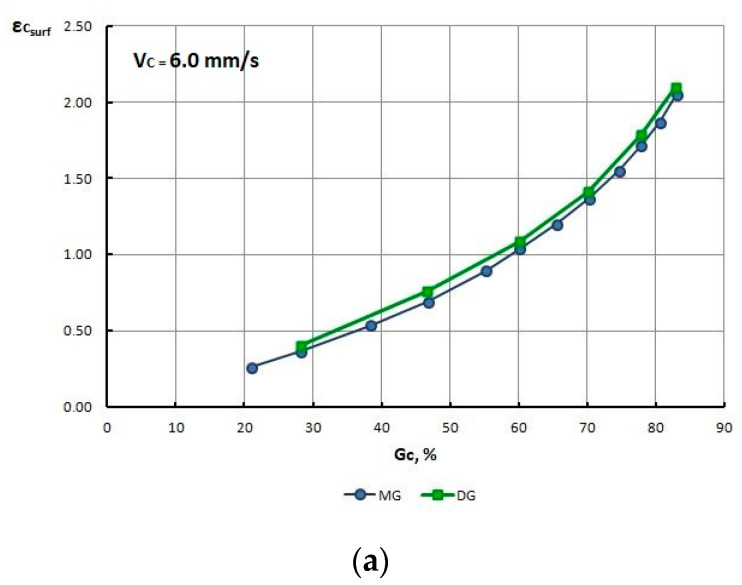

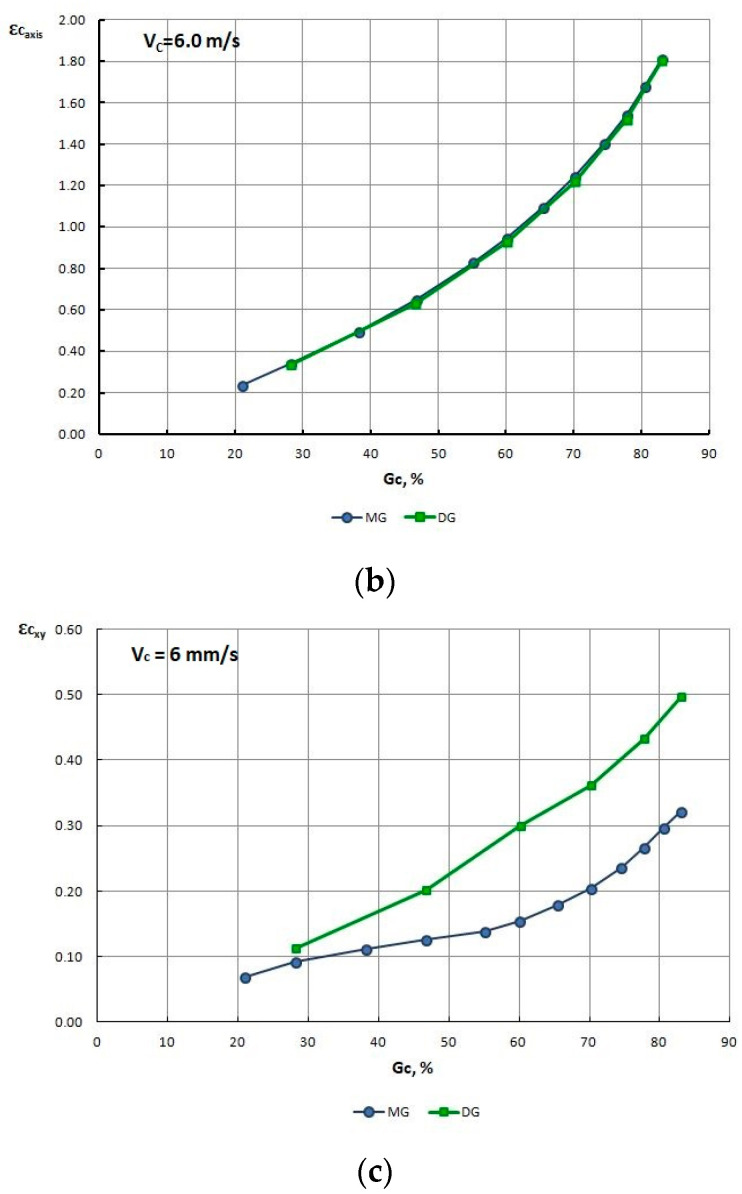
Change of strain level on the surface (**a**), axis (**b**) and non-dilatational strain (**c**) in wires drawn with low MG and high DG single section reductions, at a drawing speed of V_c_ = 6.0 m/s, as a function of the total drawing reduction G_c_%.

**Figure 15:**
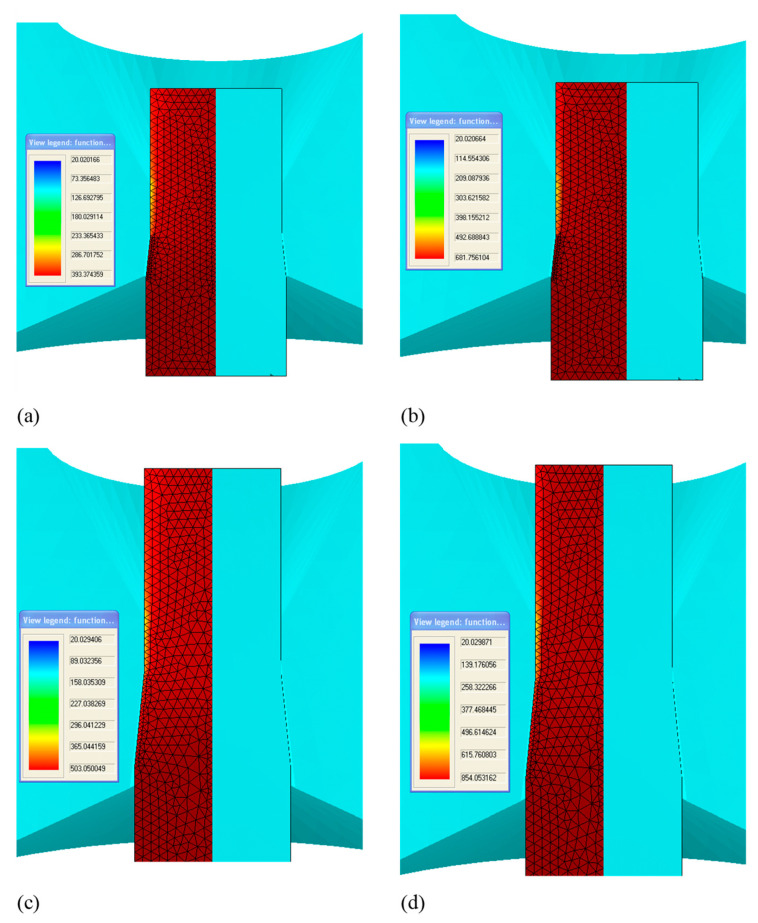
Temperature distribution within the cross-section of final wires drawn according to different drawing variants: (**a**) V_c_ = 1.6 m/s; MG (low single reductions); (**b**) V_c_ = 6.0 m/s; MG (low single reductions); (**c**) V_c_ = 1.6 m/s; DG (high single reductions); (**d**) V_c_ = 6.0 DG (high single reductions).

**Figure 16:**
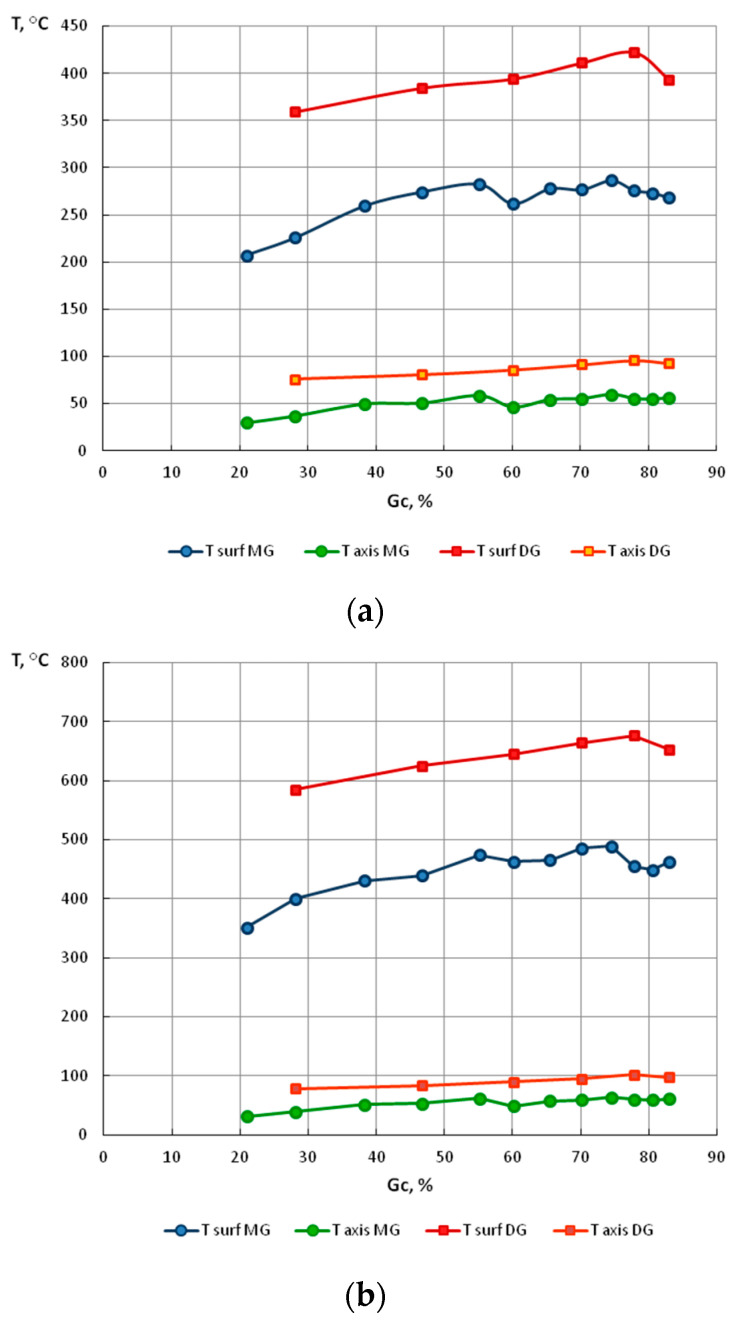
The temperature on the surface and along the axis of wires drawn with low (MG) and high (DG) single drawing reductions, at different drawing speeds, as a function of the total drawing reduction G_c_%. (**a**) V = 1.6 m/s (**b**) V = 6.0 m/s.

In order to explain this phenomenon, for all drawing variants, the authors determined the strain level and the temperature on the surface and within the axis of wires, and non-dilatational strain (redundant) on the wire surface, at the output from the gauging cylinder of the die, reading the values from these parameters from finite element mesh nodes. The test results are presented in Table 10 and Figure 11, Figure 12, Figure 13, Figure 14, Figure 15 and Figure 16.

Sample temperature distributions for final wires drawn according to different variants, obtained through modeling are shown in Figure 15.

Based on the numerical modeling results shown in Table 10 and Figure 11, Figure 12, Figure 13 and Figure 14 it can be concluded that for a drawing speed of both V_c_ = 1.6 m/s, as well as V_c_ = 6.0 m/s, the strain level on the wire surface ε_c surf_ is significantly higher than ε_c_ along their axis. However, no significant impact of the magnitude of single drawing reductions (MG and DG) on the change of these parameters was found (Figure 12 and Figure 14).

The numerical analysis of the drawing process, involving various parameters, also showed that for the process of wire drawing conducted with the scheme using large single drawing reductions, the non-dilatational strain on the wire surface after the last draw is almost twice as high as after drawing with low single drawing reductions.

However, no influence of the drawing speed on the course, as well as the value of non-dilatational strain ε_xy_ was found, since both for V_c_ = 1.6, as well as V_c_ = 6.0 m/s, the values of this parameter, do not differ significantly.

This is why the reason behind a higher R_m_ in wires drawn with low single reductions relative to the R_m_ in wires drawn with a high single reduction should be seen in the temperature of the wires drawn in different variants, the distribution of which, obtained through Drawing 2D, are shown in Figure 15, while their change, as a function of drawing process parameters, is shown in Figure 16.

The stability of retained austenite in TRIP steels depends on many factors, which include the carbon content, the austenite grain size, temperature, as well as the stress state. The specificity of retained austenite transition to martensite is dependent on three temperatures that are characteristic of TRIP steels. Below the temperature Ms, the martensite transition occurs in a spontaneous manner (a factor inducing the phase transition is exclusively the temperature drop). In the temperature range of M_s_ − M_s_^σ^, the martensite transition is caused by the stress increase, while between the temperatures M_s_^σ^ and M_d_, the phase transition of retained austenite to martensite is induced by plastic deformation [20,21]

Below the temperature M_s_, the martensitic transformation proceeds spontaneously with decreasing temperature. In the temperature interval M_s_ − M_s_^σ^, the transformation of retained austenite to martensite occurs by the effect of change in the stress state. The increase in temperature entails the need for increasing the stress necessary for the transformation to occur.

At the temperature M_s_^σ^, the stress necessary for initiating the martensitic transformation is equal to the proof stress of the retained austenite. This is a limiting value at which the transformation of retained austenite changes from the stress-induced transformation to a strain-induced one. Above the temperature M_d_, no martensitic transformation occurs, even if a plastic deformation has taken place. This is due to an increase in the stability of retained austenite at higher temperatures. The increase in temperature to values above M_d_ results in a decrease in the driving force of the transformation and the increase in the stacking fault energy, which prevents the transformation of retained austenite to martensite, only allowing its deformation.

Based on the modeling results, it can be concluded that for a drawing speed of V_c_ = 1.6 m/s, the surface temperature in wires drawn with low single reductions does not exceed 300 °C, and 420 °C for high single reductions, and decreased accordingly to approx. 50–80 °C along the wire axis, whereas for V_c_ = 6.0 m/s, the surface temperature in wires drawn with low single reductions does not exceed 480 °C, and reaches as much as 680 °C for wires drawn with high single reduction, while decreasing to ca. 50–80 °C along the axis.

It can therefore be assumed that such a large increase of the temperature on the surface of a wire drawn with high single drawing reductions, at a speed of V_c_ = 6.0 m/s, causes the heating of a subsurface wire layer of significant thickness to a temperature higher than or equal to M_d_, which blocks the transformation of retained austenite to martensite in this segment of the material, hence, a decrease in the wire’s R_m_.

In further research, we will conduct the metallographic analysis obtained after drawing process wires. The quantitative analysis of retained austenite for wires after each draw will be done for the wire surface and for the wire axis. The individual volumetric fractions of phases occurring in the structure will be calculated using three metallographic methods serving for quantitative analysis: the point-by-point method, the secant methods, and using the MET-ILO software program (version 2). The metallographic analysis can confirm the thesis that a large increase of the temperature on the surface of a wire drawn with high single drawing reductions, with high-speed of drawing causes the heating of a subsurface wire layer of significant thickness, to a temperature higher than or equal to M_d_, which blocks the transformation of retained austenite into martensite.

## 6. Conclusions

The impact of drawing process parameters on the mechanical properties and structure of the wires was researched for four variants, with two drawing speeds of 1.6 and 6.0 m/s, and two draw patterns, using low single drawing reductions (12 draws, average single reduction of approx. 13.67%) and high single drawing reductions (6 draws, average single reduction of approx. 25.53%).

Based on the conducted tests of drawn wire mechanical properties, it was concluded that for a speed of 1.6 m/s, the R_m_ and R_0.2_ of wires drawn with high single reductions were higher than these properties in wires drawn with low single reductions. In contrast, based on the analysis of the R_0.2_/R_m_ coefficient, it was concluded that wires drawn with low single reductions exhibited higher plasticity than the wires drawn with high single cross-section decrements.

It was stated that for this drawing process parameter configuration, the change trend in the mechanical properties of TRIP steel was analogous to the changes of ferrite–pearlite wire properties.

On the other hand, in the case of a drawing speed equal to 6.0 m/s, an inverse relationship was observed, i.e., higher R_m_ and R_0.2_ values for wires after a drawing process variant using low single reductions, relative to the R_m_ and R_0.2_ for wires drawn with high single reductions. In addition, a faster decline of the plasticity factor (R_0.2_/R_m_) was observed for wires drawn with low single reductions, especially in the range of 0–25% of the total reduction.

In order to explain this abnormal change in the mechanical properties of wires drawn with a speed of Vc = 6.0 m/s and with high single section decrements, a numerical analysis was conducted, which showed that for a drawing scheme with high single reductions, the non-dilatational strain on the wire surface after the last draw was almost twice as large as after a draw with low single reductions, which is a phenomenon typical for the drawing process.

However, no influence of the drawing speed on the course, as well as the value of non-dilatational strain ε_xy_ was found, since both for V_c_ = 1.6, as well as V_c_ = 6.0 m/s, the values of this parameter, do not differ significantly.

Therefore, such changes in the non-dilatational strain cannot be the reason for a higher tensile strength value in wires drawn with low single reductions, relative to the tensile strength values in wires drawn with high single reductions, at V_c_ = 6 m/s.

Based on the modeling results, it was also concluded that for a drawing speed of V_c_ = 1.6 m/s, the surface temperature in wires drawn with low single reductions did not exceed 300 °C, and 420 °C for high single reductions, and decreased accordingly to approx. 50–80 °C along the wire axis, whereas for V_c_ = 6.0 m/s, the surface temperature in wires drawn with low single reductions did not exceed 480 °C, and reached as much as 680 °C for high reduction wires, while decreasing to ca. 50–80 °C along the axis.

It was assumed that such a large increase of the surface temperature in a wire drawn with high single reductions at a speed of V_c_ = 6.0 m/s caused heating of the wire’s subsurface layer of significant thickness to a temperature higher than or equal to Md, which leads to blocking the transformation of retained austenite to martensite in this segment of the material, namely, a higher amount of retained austenite being preserved in the structure after a plastic strain, hence, a decrease in the wire’s R_m_.

## Figures and Tables

**Table 1:** Drawing parameters for various drawing speeds and single reductions.

Variant	Drawing Speed (m/s)	Number of Draws	Average Single Reduction (%)	Total Reduction (%)	Process Conditions
A	1.6	12	13.67	82.97	Laboratory conditions
B	1.6	6	25.53	82.97	Laboratory conditions
C	6.0	12	13.67	82.97	Industrial conditions
D	6.0	6	25.53	82.97	Industrial conditions

**Table 2:** Scheme of applied single drawing reductions and the total drawing reduction in the drawing process involving TRIP steel wire rod, in a system with low single reductions.

Draw No.	ϕ (mm)	G_p_ (Single Reduction) (%)	G_c_ (Total Reduction) (%)
0	6.30	-	-
1	5.60	20.99	20.99
2	5.34	9.07	28.15
3	4.95	14.07	38.27
4	4.60	13.64	46.69
5	4.22	15.84	55.13
6	3.98	11.05	60.09
7	3.70	13.58	65.51
8	3.44	13.56	70.18
9	3.18	14.55	74.52
10	2.97	12.77	77.78
11	2.78	12.39	80.53
12	2.60	12.53	82.97

**Table 3:** Scheme of applied single drawing reductions and the total drawing reduction in the drawing process involving TRIP steel wire rod, in a system with high single reductions.

Draw No.	ϕ (mm)	G_p_ (Single Reduction) (%)	G_c_ (Total Reduction) (%)
0	6.30	-	-
1	5.34	28.15	28.15
2	4.60	25.80	46.69
3	3.98	25.14	60.09
4	3.44	25.29	70.18
5	2.97	25.46	77.78
6	2.60	23.36	82.97

**Table 4:** Results of tests regarding the mechanical properties of TRIP steel wires drawn as per variant A (V_c_ = 1.6 m/s, low single drawing reductions).

Wire ϕ (mm)	R_m_ (MPa)	R_0.2_ (MPa)	R_0.2_/R_m_
6.30	929.00	481.00	0.52
5.60	1182.00	845.00	0.71
5.34	1278.00	943.00	0.74
4.95	1396.00	1070.00	0.77
4.60	1492.00	1226.00	0.82
4.22	1560.00	1362.00	0.87
3.98	1605.00	1439.00	0.90
3.70	1663.00	1507.00	0.91
3.44	1697.00	1561.00	0.92
3.18	1891.00	1748.00	0.92
2.97	1920.00	1783.00	0.93
2.78	1948.00	1834.00	0.94
2.60	1986.00	1885.00	0.95

**Table 5:** Results of tests regarding the mechanical properties of TRIP steel wires drawn as per variant B (V_c_ = 1.6 m/s, high single drawing reductions).

Wire ϕ, (mm)	R_m_, (MPa)	R_0.2_, (MPa)	R_0.2_/R_m_
6.30	929.00	481.00	0.52
5.34	1381.00	1190.00	0.86
4.60	1586.00	1442.00	0.91
3.98	1709.00	1574.00	0.92
3.44	1793.00	1699.00	0.95
2.97	2005.00	1911.00	0.95
2.60	2112.00	2032.00	0.96

**Table 6:** Results of tests regarding the mechanical properties of TRIP steel wires drawn as per variant C (V_c_ = 6.0 m/s, low single drawing reductions).

Wire ϕ (mm)	R_m_ (MPa)	R_0.2_ (MPa)	R_0.2_/R_m_
6.30	929.00	481.00	0.52
5.60	1360.00	1123.00	0.83
5.34	1466.00	1211.00	0.83
4.95	1607.00	1318.00	0.82
4.60	1703.00	1446.00	0.85
4.22	1795.00	1470.00	0.82
3.98	1832.00	1525.00	0.83
3.70	1909.00	1613.00	0.84
3.44	1957.00	1659.00	0.85
3.18	2161.00	1785.00	0.83
2.97	2193.00	1835.00	0.84
2.78	2221.00	1902.00	0.86
2.60	2254.00	1943.00	0.86

**Table 7:** Results of tests regarding the mechanical properties of TRIP steel wires drawn as per variant D (V_c_ = 6.0 m/s, high single drawing reductions).

Wire ϕ (mm)	R_m_ (MPa)	R_0.2_ (MPa)	R_0.2_/R_m_
6.30	929.00	481.00	0.52
5.34	1405.00	1180.00	0.84
4.60	1632.00	1387.00	0.85
3.98	1781.00	1476.00	0.83
3.44	1908.00	1591.00	0.83
2.97	2142.00	1801.00	0.84
2.60	2186.00	1912.00	0.87

**Table 8:** Function parameters (1) approximating the results of plasticometric tests for TRIP steel.

a_1_	a_2_	a_3_	a_4_	a_5_	a_6_	a_7_	a_8_	a_9_
847.391	−0.0783	−0.0137	−0.0484	−0.0061	−0.0663	−0.0003	−0.0405	−0.00093

**Table 9:** Comparison of tensile strength test results for TRIP steel wires drawn with different speeds (V_c_ = 1.6 m/s and V_c_ = 6.0 m/s), and low (MG) and high (DG) single drawing reductions, determined through numerical modeling and an experiment.

	R_m_, (MPa)			
	Low Single Reductions		High Single Reductions	
	V_c_ = 1.6 (m/s)			
**Wire ϕ, mm**	**Numerical Modeling**	**Experiment**	**Numerical Modeling**	**Experiment**
5.6	1123.3	1182		
5.34	1258	1278	1348.2	1381
4.95	1345.6	1396		
4.6	1437.6	1492	1526.7	1586
4.22	1533.1	1560		
3.98	1602.9	1605	1674.1	1709
3.7	1664.8	1663		
3.44	1731.1	1697	1760.6	1793
3.18	1792.2	1891		
2.97	1851.2	1920	1926.5	2005
2.78	1901.7	1948		
2.6	1950	1986	2048.2	2112
	**R_m_, (MPa) V_c_ = 6.0 (m/s)**			
5.6	1228.6	1360		
5.34	1367.6	1466	1249.9	1405
4.95	1460.4	1607		
4.6	1557.2	1703	1494.3	1632
4.22	1658.4	1795		
3.98	1733.3	1832	1685.6	1781
3.7	1799.8	1909		
3.44	1870.2	1957	1835.4	1908
3.18	1934.8	2161		
2.97	1997.4	2193	1945	2142
2.78	2051.5	2221		
2.6	2103.4	2254	2070.2	2186

**Table 10:** The strain level on the wire surface and axis, non-dilatational strain and the temperature determined through a theoretical analysis of the drawing process with low (MG) and high (DG) single section reductions and at various drawing speeds (V_c_ = 1.6 m/s and V_c_ = 6.0 m/s).

V_c_ = 1.6 (m/s)										
	MG (Low Single Reductions)					DG (High Single Reductions)				
Wire ϕ, mm	ε_c surf_	ε_c axis_	ε_xy_	Temperature (°C)		ε_c surf_	ε_c axis_	ε_xy_	Temperature (°C)	
				Surface	Axis				Surface	Axis
5.60	0.2521	0.2300	−0.0634	207.10	29.70					
5.34	0.3595	0.3180	−0.0875	225.70	36.90	0.3605	0.3220	−0.1220	359.20	76.00
4.95	0.5171	0.4680	−0.1067	259.30	49.30					
4.60	0.6580	0.6080	−0.1172	274.20	50.30	0.6790	0.6080	−0.2170	384.30	80.60
4.22	0.8395	0.7750	−0.1260	282.60	58.20					
3.98	0.9620	0.8830	−0.1370	261.80	46.20	0.9820	0.8900	−0.2980	394.10	85.70
3.70	1.1140	1.0190	−0.1680	277.70	54.01					
3.44	1.2640	1.1550	−0.1880	276.70	55.10	1.2940	1.1660	−0.3770	411.10	91.30
3.18	1.4350	1.3010	−0.2210	286.40	59.40					
2.97	1.5800	1.4280	−0.2480	276.10	55.30	1.6050	1.4440	−0.4590	422.30	95.80
2.78	1.7090	1.5490	−0.2740	273.20	54.50					
2.60	1.8610	1.6700	−0.3010	268.10	56.10	1.8910	1.6900	−0.5340	393.50	92.70
**V_c_ = 6.0 (m/s)**										
5.60	0.2597	0.2340	−0.0694	350.90	31.60					
5.34	0.3657	0.3420	−0.0920	400.10	38.70	0.4020	0.3340	−0.1130	585.10	78.30
4.95	0.5355	0.4970	−0.1112	430.90	51.60					
4.60	0.6940	0.6480	−0.1254	439.10	52.80	0.7592	0.6320	−0.2020	625.30	83.40
4.22	0.8940	0.8280	−0.1380	474.40	61.80					
3.98	1.0370	0.9470	−0.1540	462.50	48.90	1.0830	0.9280	−0.3000	645.00	89.30
3.70	1.1980	1.0960	−0.1790	465.90	57.60					
3.44	1.3700	1.2450	−0.2040	485.30	59.30	1.4090	1.2190	−0.3620	664.60	95.20
3.18	1.5470	1.4040	−0.2356	489.50	63.70					
2.97	1.7140	1.5430	−0.2667	455.40	59.60	1.7890	1.5140	−0.4330	676.30	101.90
2.78	1.8710	1.6770	−0.2973	448.80	58.70					
2.60	2.0510	1.8110	−0.3218	461.80	60.50	2.1010	1.8000	−0.4970	653.20	97.50
